# Calendar

**DOI:** 10.1155/S1463924688000306

**Published:** 1988


### Calendar

### JUNE

5–11 June 1988 *Achema 88 – Chemical Engineering Exhibition & Congress: Frankfurt, FR Germany.* Contact Dechema, Postfach 970146, D6000 Frankfurt/Main 97, FR Germany.

### SEPTEMBER

5–9 September 1988 *Analytical Plasma Spectrometry: Loughborough, UK.* Contact Mrs J. Stirling, Department of Chemistry, Loughborough University of Technology, Loughborough, Leicestershire LE11 3TU, UK.

12 September 1988 *Atomic Absorption Spectrometry: Loughborough, UK.* Contact Mrs J. Stirling (as above).

14–16 September 1988 *Modern Electroanalytical Methods: Loughborough, UK.* Contact Mrs J. Stirling (as above).

19–23 September 1988 *Microcomputers in the Laboratory: Loughborough, UK.* Contact Mrs J. Stirling (as above).

### OCTOBER

11–13 October 1988 *The British Laboratory Week: Grand Hall, Olympia, London.* Contact Curtis Steadman & Partners Ltd, The Hub, Emson Close, Saffron Walden, Essex CB10 1HL, UK.

### NOVEMBER

2–4 November 1988 *Analysis, Information and Quality: Analytical Information its Quality, Relevance and Costs: Sutton Coldfield, UK.* Contact Mr R. Lidgett, Fron Cottage, Lladynan, Llangollen, Clwyd, Wales LL20 7AJ, UK.

9–12 November 1988 *Clinica-Lab/Medic-Asia: World Trade Centre, Singapore.* Contact Curtis Steadman & Partners, Saffron Walden (as above).

### 1989

30 July to 5 August 1989 *SAC 89: Cambridge, UK.* Contact Analytical Division, Royal Society of Chemistry, Burlington House, London W1V 0BN.

25–28 September 1989 *Third International Symposium on Kinetics in Analytical Chemistry: Dubrovnik, Yugoslavia.* Contact Professor Gordana A. Milonović, Department of Chemistry, University of Belgrade, KAC PO Box 550, 11001 Belgrade, Yugoslavia.

### NOTES FOR AUTHORS

*Journal of Automatic Chemistry* covers all aspects of automation and mechanization in analytical, clinical and industrial environments. The *Journal* publishes original research papers; short communications on innovations, techniques and instrumentation, or current research in progress; reports on recent commercial developments; and meeting reports, book reviews and information on forthcoming events. All research papers are refereed.

### Manuscripts

Two copies of articles should be submitted. All articles should be typed in double spacing with ample margins, on one side of the paper only. The following items should be sent: (1) a title-page including a brief and informative title, avoiding the word 'new' and its synonyms; a full list of authors with their affiliations and full addresses; (2) an abstract of about 250 words; (3) the main text; (4) appendices (if any); (5) references; (6) tables, each table on a separate sheet and accompanied by a caption; (7) illustrations (diagrams, drawings and photographs) numbered in a single sequence from 1 upwards and with the author's name on the back of every illustration; captions to illustrations should be typed on a separate sheet. Papers are accepted for publication on condition that they have been submitted only to this *Journal.*

### References

References should be indicated in the text by numbers following the author's name, i.e. Skeggs [6]. In the reference section they should be arranged thus:

to a journal

Manks, D. P., *Journal of Automatic Chemistry,* **3** (1981), 119.

to a book

Malmstadt, H. V., in *Topics in Automatic Chemistry,* Ed. Stockwell, P. B. and Foreman, J. K. (Horwood, Chichester, 1978), p. 68.

### Illustrations

Original copies of diagrams and drawings should be supplied, and should be drawn to be suitable for reduction to the page or column width of the *Journal,* i.e. to 85 mm or 179 mm, with special attention to lettering size. Photographs may be sent as glossy prints or as negatives.

### Proofs and offprints

The principal or corresponding author will be sent proofs for checking and will receive 50 offprints free of charge. Additional offprints may be ordered on a form which accompanies the proofs.

*Manuscripts should be sent to Dr P. B. Stockwell, P.S. Analytical Ltd, Arthur House, Cray Avenue, Orpington, Kent BR5 3TR, UK.*

